# Effects of X‐Ray Irradiation on the Biological Parameters, Gut Microbiota, and Gene Expression of *Bactrocera dorsalis*: Implications for the Sterile Insect Technique

**DOI:** 10.1111/eva.70158

**Published:** 2025-10-02

**Authors:** Jia Lin, Wensha Ding, Jun Chen, Guoqing Yue, Bo Wang, Qing'e Ji

**Affiliations:** ^1^ Institute of Biological Control Fujian Agriculture and Forestry University Fuzhou China; ^2^ Key Laboratory of Biopesticide and Chemical Biology Ministry of Education Fuzhou China; ^3^ State Key Laboratory of Ecological Pest Control for Fujian and Taiwan Crops Fuzhou China

**Keywords:** *Bactrocera dorsalis*, gut bacteria, male quality, sterile insect technique, transcriptome, X‐ray

## Abstract

The sterile insect technique (SIT), traditionally reliant on gamma irradiation, has been an effective strategy for controlling *Bactrocera dorsalis* . However, strict regulations governing gamma radiation sources and the limited research on the responses of *B. dorsalis* to X‐ray irradiation have hindered the further development of SIT. This study demonstrated that X‐ray dosage, pupal age, and their interaction significantly influenced the emergence parameters of *B. dorsalis* . Further experiments revealed that irradiating 8‐day‐old pupae resulted in a significant reduction in flight ability, lifespan, and fecundity in emerging adults. However, optimized doses ranging from 70 to 100 Gy effectively induced complete sterility while exerting minimal adverse effects on male quality. X‐ray irradiation induced notable shifts in the gut microbiota composition of *B. dorsalis* , marked by a reduction in the abundance of *Enterobacter*, *Citrobacter*, and *Proteus*, accompanied by an enrichment of *Providencia*. Additionally, broad correlations among dominant bacterial genera were observed. Transcriptomic analysis further indicated that irradiation had a profound impact on gene expression in both male and female adults, with 100 and 34 differentially expressed genes (DEGs) identified in females and males, respectively. Gene Ontology (GO) enrichment analysis revealed six enriched GO terms common to both sexes. Correlation analysis suggested potential associations between specific differentially abundant bacterial genera and DEGs. These findings optimize X‐ray‐based SIT for *B. dorsalis* and provide new insights into its effects on gut microbiota and gene expression, offering theoretical support for the refinement of SIT strategies.

## 1 Introduction

*Bactrocera dorsalis* (Diptera: Tephritidae) poses a significant threat to both the quality and yield of host crops, as well as the sustainable development of the global horticulture industry (Cai et al. 2017; Liu et al. 2019; Mutamiswa et al. 2021). Traditionally, chemical control has been the most widely used and effective strategy for managing this pest. While pesticide applications can rapidly suppress pest populations to some extent, the prolonged and excessive use of pesticides has led to serious issues such as pest resistance and environmental pollution (Jin et al. 2011; Cai et al. 2018a; Lin et al. 2022). Thus, there is an imperative to implement more environmentally friendly and sustainable management strategies.

The sterile insect technique (SIT) is recognized for its eco‐friendliness and high species specificity, making it one of the most effective alternatives to chemical control methods (Vargas et al. 2015; Lin et al. 2020). This technique generally involves sterilizing mass‐reared male insects with ionizing radiation, followed by the inundative introduction of irradiated male agents into wild target populations, resulting in sterile matings producing unviable offspring (Mau et al. 2007; Vreysen and Robinson 2011). In the context of tephritid fruit flies, numerous programs have integrated SIT into area‐wide management strategies for economically significant species across various regions to achieve objectives such as prevention, suppression, and eradication (Vargas et al. 2015; Cai et al. 2018a, 2018b; Cai et al. 2018). Traditionally, sterile fruit flies are generated by subjecting pupae to gamma rays from radioisotopes like cobalt‐60 (Co^60^) or cesium‐137 (Cs^137^) (Mastrangelo et al. 2010; Pereira et al. 2013). However, issues related to the availability, cost, and safety of radioisotopes have constrained the broader application and adoption of gamma radiation in SIT programs (Mastrangelo et al. 2010). Recently, X‐rays have emerged as a promising alternative radiation source for the SIT of various agricultural pests (Yun et al. 2016; Zhao et al. 2022; Zhang et al. 2023; Huang et al. 2024). This shift is largely due to their advantages, including lower transport costs, easier accessibility, and the absence of radioactive waste (Yamada et al. 2014).

Although the SIT program for *B. dorsalis* has been developed over a long period and has achieved remarkable success globally, gamma radiation has traditionally been the primary radiation source (Ji et al. 2013; Cai et al. 2018b; Yusof et al. 2019). Given the exceptional merits of X‐ray, two key factors justify its adoption: (1) the short shelf‐life of sterile agents restricts their effective transport from mass‐rearing facilities (e.g., in Hawaii and Southeast Asia) to remote areas, leading to the unavailability of sterile *B. dorsalis* (Steck et al. 2019; Fezza et al. 2021); and (2) the relatively slow progress of SIT programs in many regions, due to strict regulations on radioisotopes and security concerns, poses a significant challenge, particularly in areas experiencing immense pressure from *B. dorsalis* infestations (Mutamiswa et al. 2021). Therefore, a thorough investigation into the use of X‐ray as a radiation source is essential, with a focus on identifying the optimal X‐ray dose and the preferred pupal age of *B. dorsalis* for irradiation, as well as evaluating the effects of X‐ray treatment on key biological traits in adults.

Moreover, the effects of ionizing radiation extend beyond immediate physiological impacts on the organism; they may also influence the regulation of the insect's gut microbiota and gene expression levels (Zhang, Cai, et al. 2021; Zhang, Chen, et al. 2021; Jiang 2023; Singh et al. 2024). Accumulating evidence indicates that gut symbionts are integral to multiple physiological processes in tephritid species, such as development, immunity, reproduction, and metabolism, and also facilitate their success in biological invasions (Hassan et al. 2020; Noman et al. 2021; Hafsi and Delatte 2023). Additionally, progress in RNA sequencing and bioinformatics has broadened our understanding of signaling and metabolic pathways associated with gene function in insect species (Li et al. 2021). Therefore, examining the effects of X‐ray exposure on the gut microbiome and transcriptomic profile of *B. dorsalis* could offer crucial insights into the mechanisms underlying its resistance to radiation stress, aiding in the optimization of SIT programs.

To explore the potential of X‐ray irradiation as a practical alternative to gamma rays in SIT programs against *B. dorsalis* , this study systematically evaluated the biological and physiological responses of *B. dorsalis* to X‐ray exposure. We hypothesized that the X‐ray irradiation would influence not only emergence and adult performance, but also physiological regulation at the microbial and transcriptomic levels. Accordingly, we assessed emergence and biological parameters, and examined potential disruptions in gut microbial communities and gene expression patterns in both sexes. By integrating biological, microbiological, and transcriptomic data, this work aims to provide a comprehensive framework for optimizing X‐ray‐based sterilization protocols in the management programs of *B. dorsalis* .

## 2 Materials and Methods

### 2.1 Insects

The *B. dorsalis* genetic sexing strain (GSS) was derived from a pupal color sexing strain established in Hawaii and was introduced to Fuzhou in 2007. In this strain, laboratory‐reared female pupae are white, whereas male pupae are brown (Ji et al. 2007). To enhance adaptability, the strain was hybridized and backcrossed with wild Chinese *B. dorsalis* females (Ji et al. 2007). The *B. dorsalis* GSS was maintained under controlled environmental conditions (25°C ± 1°C, 65% ± 5% RH, and a 12:12 light: dark photoperiod). It was screened each generation to ensure the stability of its sexing mechanism (Fezza et al. 2018). Wild *B. dorsalis* was obtained from infested mangoes in an orchard located in Fuqing, Fujian. These collected infested mangoes were reared on sand under the same controlled environmental conditions, and the resulting pupae harvested.

Both wild and GSS adults were kept in mesh cages (30 × 30 × 30 cm) and provided with a food mixture of yeast extract and sugar in a 1:3 proportion, along with wet cotton for water. The eggs of wild *B. dorsalis* were collected by puncturing mango fruits, and the larvae were reared on an artificial diet, as Chang et al. (2006) described. The pupae were then harvested, and the emerging progeny of wild *B. dorsalis* were subsequently used in mating trials.

### 2.2 Effects of Irradiation on the Emergence Parameters of *B. dorsalis*

The emergence parameters of *B. dorsalis* were evaluated by subjecting them to different doses of X‐ray doses (50, 100, 150, 200, 250, and 300 Gy) at various pupal ages (5, 6, 7, 8, and 9 days). Fifty white (female) or brown (male) pupae were individually selected 1 day prior to irradiation. The irradiation procedure was conducted using an X‐ray irradiator (6150AD; Nuctch Co. Ltd) at the dosage rate of 1 Gy/min. After treatment, irradiated pupae were transferred into Petri dishes (9 cm in diameter). The emergence of flies was monitored daily and categorized into normal adults, deformed adults, or partial emergence. The experiment was conducted under controlled conditions (25°C ± 1°C, 65% ± 5% RH) with five replicates per treatment.

### 2.3 Effects of Irradiation on the Flight Ability of *B. dorsalis*

After irradiation exposure, one hundred brown or white pupae were placed in a 9 cm Petri dish, which was then positioned inside a black cylindrical tube (inner diameter: 10 cm; height: 10 cm). The interior wall of the tube was coated with talc powder to prevent adults from climbing out. The tube, along with the Petri dish, was placed inside a mesh cage, with 30 W fluorescent lamps positioned 20 cm above. The experiment was conducted under controlled conditions (25°C ± 1°C, 65% ± 5% RH). Flies that emerged and flew out of the tube were gathered every 12 h until all had died. Flight capability was determined by the ratio of (flies that flew out of the tube/total number of pupae) × 100. Six replicates were conducted for irradiated females (IF), unirradiated females (UF), irradiated males (IM), and unirradiated males (UM).

### 2.4 Effects of Pupal Irradiation on the Longevity and Fecundity of Emerged *B. dorsalis*

Newly emerged adults were paired and transferred into a rearing device (Lin et al. 2023). In total, 20 paired adults were tested for each treatment and control group. Water and adult food were provided throughout the experiment. Filter paper absorbed with orange juice was placed into an oviposition tube with evenly spaced holes on the surface. Oviposition tubes were provided to allow females to lay eggs. Daily records were kept of the number of dead flies and eggs laid. This trial was carried out in a regulated environment (25°C ± 1°C, 65% ± 5% RH).

### 2.5 Effects of Irradiation on the Sterile Rate of *B. dorsalis* Males

Preliminary experiments indicated that exposing 8‐day‐old *B. dorsalis* pupae to X‐ray doses exceeding 100 Gy led to complete egg sterilization. Consequently, further experiments were conducted using irradiation doses between 50 and 100 Gy for the 8‐day‐old pupae. One hundred newly emerged IM and 100 newly emerged UF were introduced into a test cage. Water and adult food were provided throughout the experiment. A filter paper soaked in orange juice was placed inside a 50 mL centrifuge tube with evenly distributed small holes on its surface to facilitate egg laying. Females were allowed to lay eggs for 1 h when they were 12, 16, and 20 days old. A total of 500 eggs from each cage were randomly selected and placed on filter paper, misted with sterile water to prevent dehydration. Egg hatchability was recorded daily for five consecutive days. The sterile rate of eggs was calculated by the formula: (number of eggs hatched/total number of eggs) × 100. This trial was conducted in a regulated environment (25°C ± 1°C, 65% ± 5% RH) with six replicates.

### 2.6 Effects of Irradiation on the Mating Competitiveness of *B. dorsalis* Males

Upon emergence, the IM and wild *B. dorsalis* were reared separately in cages until they reached sexual maturity. All adults used in the experiment were between 15 and 20 days old and remained virgins. One hour before the experiment, 50 IM, 50 wild males, and 50 wild females were transferred into a test cage, placed outdoors to acclimate to the environment. The thoraxes of irradiated or wild males were marked with non‐toxic dyes, and dye colors were alternated between replicates to eliminate bias. The experiment began 2 h before sunset and continued until 30 min after sunset. Copulating pairs were gently collected using a tube at 10‐min intervals, and the copulating males were then identified as either wild or irradiated. The Relative Sterility Index (RSI) was calculated as the number of IM that mated with wild females divided by the total number of mating events (including those involving both treated and wild males) (Zhang, Cai, et al. 2021; Zhang, Chen, et al. 2021). This bioassay was conducted with six replicates.

### 2.7 Effects of Irradiation on the Gut Bacteria Communities of *B. dorsalis*

Sixty newly emerged irradiated and unirradiated *B. dorsalis* (both males and females) were dissected according to the method described by Lin et al. (2023). The midgut and hindgut were obtained for subsequent DNA extraction. Genomic DNA was isolated using the E.Z.N.A Mag‐Bind Soil DNA Kit (Omega Bio‐Tek, USA). The V3–V4 variable region of the bacterial 16S rRNA gene was amplified using the primer pair 341F (5′‐CCTACGGGNGGCWGCAG‐3′) and 805R (5′‐GACTACHVGGGGTATCTAATCC‐3′). The PCR amplicons were assessed for library size via 2% agarose gel electrophoresis, and library concentration was measured using a Qubit 3.0 fluorometer (Thermo Fisher Scientific, USA). Ultimately, the library was subjected to sequencing on an Illumina MiSeq PE300 system (Illumina MiSeq, USA). Three replications were performed for each sex.

For the analysis of 16S rDNA sequences, adapter sequences were removed from the raw data using the Cutadapt tool (v1.18) (Martin 2011). The paired‐end reads were combined by exploiting overlapping regions using PEAR software (v0.9.8) (Zhang et al. 2014), and sequence quality was checked using PRINSEQ (v0.20.4) (Schmieder and Edwards 2011). The Usearch tool (v11.00.667) was employed to define operational taxonomic units (OTUs) (Edgar 2013). The sequences were clustered into OTUs with a 100% similarity cutoff, and taxonomic assignments were made using the GTDB database (Edgar 2018). Alpha diversity was calculated with the Mothur software (v1.43.0) (Schloss et al. 2009), while beta diversity was assessed and visualized using the phyloseq (v1.30.0) and vegan packages (v2.5) in R (v3.6.0) (McMurdie and Holmes 2013). Differentially abundant bacterial genera (DABGs) were identified using DESeq2 (v1.26.0) with a *p*‐value < 0.05 and |FoldChange| > 1. Additionally, the correlation analysis of dominant bacterial genera (relative abundance > 1%) of both sexes of *B. dorsalis* was estimated by Pearson's correlation coefficient, and the correlation was visualized by circus plots (Li et al. 2022).

### 2.8 Effects of Irradiation on the Transcriptome of *B. dorsalis*

A total of 10 newly emerged *B. dorsalis* males or females were collected, and three replications were performed for each sex. RNA from *B. dorsalis* was isolated with the assistance of DP431 kits (Tiangen Biotech, Beijing, China). The quality and concentration of RNA were evaluated using a micro‐ultraviolet spectrophotometer (SMA4000, Merinton, British). Subsequently, the RNA libraries were constructed using the protocol of reverse transcription kits (Fermentas, USA). Finally, cDNA libraries were sequenced on the Illumina HiSeq platform using a paired‐end 150 bp strategy.

Raw data were processed using Trimmomatic (v0.36) to remove adapters, poly‐N sequences, and low‐quality reads (Bolger et al. 2014). Quality‐controlled sequences were then aligned to the reference genome (NCBI accession number: ASM2337382v1) using HISAT2 (v2.1.0) (Kim et al. 2015), and the mapping results were assessed with RseQC (v2.6.1) (Wang et al. 2012). After mapping, gene expression levels were quantified as Transcripts Per Million (TPM) using StringTie (v1.3.3b) (Pertea et al. 2015). Differentially expressed genes (DEGs) were identified using DESeq2 (v1.26.0) with a *q*‐value < 0.05 and |fold change| > 2. Functional enrichment analysis of DEGs was performed based on Gene Ontology (GO) terms using clusterProfiler (v3.0.5) in R (v3.6.0) (Yu et al. 2012; Love et al. 2014). To confirm the reliability of the transcriptome data, reverse transcription‐quantitative PCR (RT‐qPCR) was performed. The DEGs and the primers used for RT‐qPCR are listed in Table S1. The *α‐Tubin* (GenBank accession no: GU269902) of *B. dorsalis* was used as a control.

### 2.9 Correlation Analysis of 16S rRNA Gene Sequencing and Transcriptomic Data

The similarity between bacterial and transcriptome samples was assessed using Procrustes analysis, where residuals represented the degree of similarity. The *p*‐value was determined through a permutation test based on the sum of squares of these residuals. DABGs and DEGs were correlated using Pearson's correlation coefficient. The correlations were then visualized using circos plots.

### 2.10 Data Analysis

Data analysis was performed using the SPSS software (v24.0). The normal distribution and variance homogeneity of the data were first verified. Arcsine square‐root transformation was performed for the percentage data, whereas untransformed data were presented in tables and figures.

The effects of pupal age, sex, irradiation dose, and their interactions on the emergence parameters of *B. dorsalis* were evaluated using a three‐way ANOVA. One‐way ANOVA was utilized to evaluate the impacts of irradiation on the fecundity and mating competition of *B. dorsalis* . Differences between treatments and the control group in terms of emergence parameters, flight ability, longevity, fecundity, sterility rate, and mating competitiveness were assessed using Tukey's multiple comparison test. An independent samples *t*‐test was conducted to analyze the difference between the control and treatment groups with regard to the relative abundance of bacterial orders or genera. Differences in richness and diversity indices between the control and treatment groups were assessed using independent samples *t*‐tests. PERMANOVA and PERMDISP analyses were performed on bacterial and transcriptomic datasets using the vegan package (v2.5) in R (v3.6.0). Circos plots illustrating Pearson correlation relationships were generated using the circlize (v0.4.16), RColorBrewer (v1.1.3), and gridBase (v0.4.7) packages. Circos plots depicting the associations among samples, treatments, and bacterial genera were created using reshape2 (v1.4.4) and circlize (v0.4.16). Heat tree visualizations were constructed with phyloseq (v1.30.0) and metacoder (v0.3.5). Manhattan plots were generated using ggplot2 (v3.5.1) and dplyr (v1.1.4). Gene Ontology (GO) enrichment results were visualized using circlize (v0.4.16) and grid (v4.0.4).

## 3 Results

### 3.1 Effects of Irradiation on the Emergence Parameters of *B. dorsalis*

**FIGURE 1:**
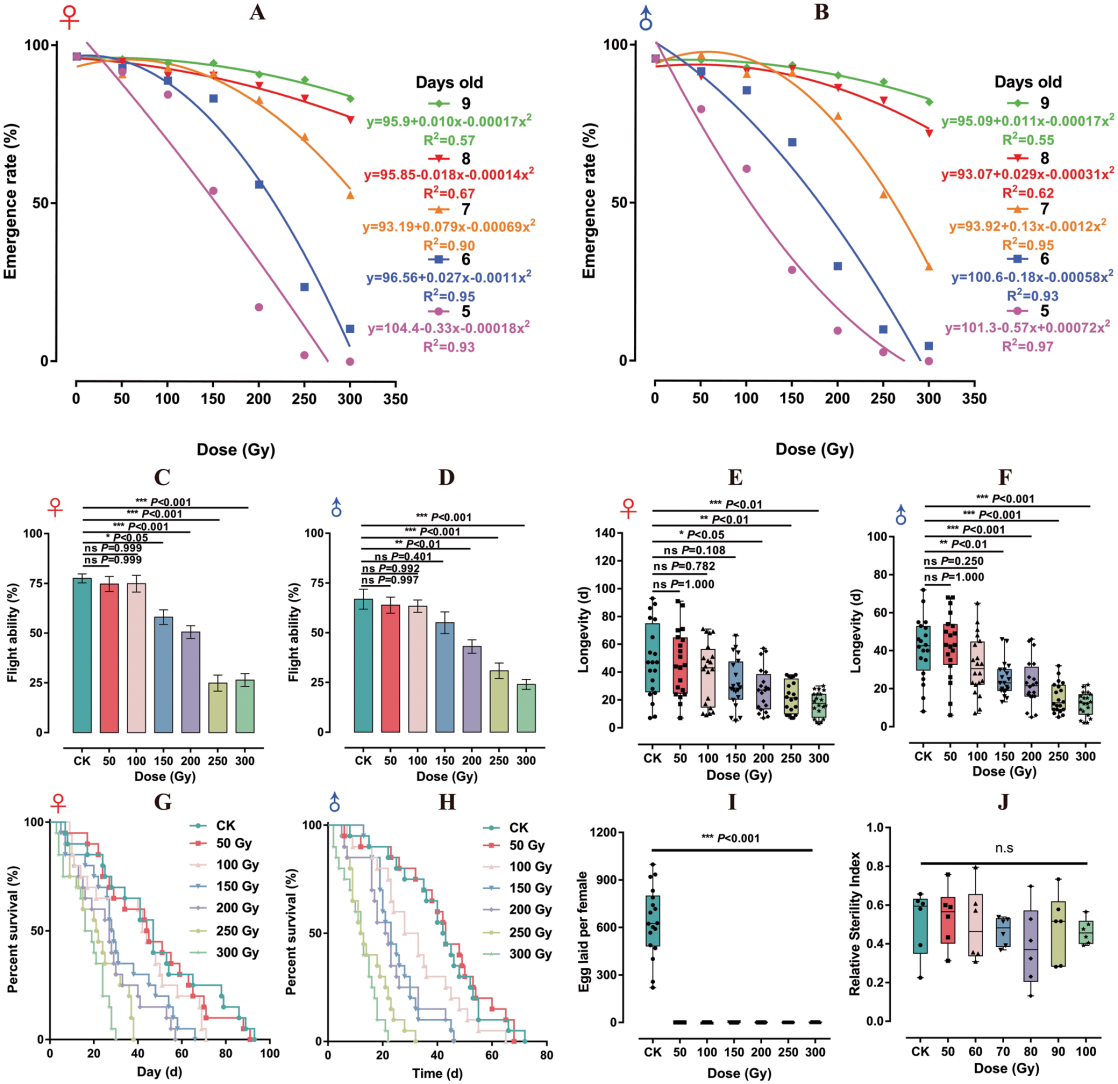
Effect of pupal X‐ray irradiation on the *Bactrocera dorsalis* . Emergence rate of female (A) and male (B) subjecting to different X‐ray doses at various pupal ages. Flight ability (C: Female; D: Male), longevity (E: Female; F: Male), survival curve (G: Female; H: Male), fecundity (I) and relative sterility index (J) of *B. dorsalis* which were subjected to irradiation at the pupal stage (8 days old). The flight ability data (C: Female; D: Male) were presented as the mean ± standard error (SE), whereas the longevity (E: Female; F: Male), fecundity (I), and relative sterility index (J) data were visualized using box plots with whiskers. Significant differences between treatments and the control group were determined using Tukey's multiple comparison test.

A three‐way ANOVA revealed that all main factors and their interactions significantly impacted the emergence of *B. dorsalis* , except for the interaction between sex and irradiation dose (Table S2). A significant dose‐dependent effect was observed in 5‐ and 6‐day‐old pupae of both sexes. In 7‐day‐old pupae, irradiation doses above 200 Gy led to a markedly reduced emergence rate compared to the control group for both females (200 Gy: *p* < 0.01; 250 Gy: *p* < 0.01; 300 Gy: *p* < 0.01) and males (200 Gy: *p* < 0.01; 250 Gy: *p* < 0.01; 300 Gy: *p* < 0.01). Similarly, 8‐day‐old pupae exposed to doses of 250 Gy (female: *p* < 0.05; male: *p* < 0.05) and 300 Gy (female: *p* < 0.05; male: *p* < 0.01), as well as 9‐day‐old pupae exposed to 300 Gy (female: *p* < 0.05; male: *p* < 0.01), showed a remarkable reduction in the number of adults emerged. Furthermore, significant age‐dependent effects were detected when the irradiation dose exceeded 100 Gy for male pupae and 200 Gy for female pupae. No significant differences were observed between 8‐day‐old and 9‐day‐old pupae, regardless of irradiation dose or sex (Figure 1A,B; Tables 1 and 2). The results pertaining to the partial emergence rate (Table S3) and deformed adult rate (Table S4) are presented in Appendix S1.

**TABLE 1:** Effects of different age of pupae subjected to X‐ray irradiation on the emergence parameters of *Bactrocera dorsalis* females (one‐way ANOVA).

Emergence rate (%)					
Dose (Gy)	Age (days)				
	5	6	7	8	9
50	91.60 ± 1.47aAB	92.80 ± 1.74aAB	90.80 ± 1.36aAB	94.80 ± 1.36aA	95.60 ± 0.40aAB
100	84.40 ± 3.31aB	88.80 ± 2.65aAB	92.80 ± 1.85abA	90.40 ± 3.31aAB	94.40 ± 0.40aAB
150	54.00 ± 2.53cC	83.20 ± 2.87bB	91.20 ± 1.20aA	90.40 ± 1.17abABC	94.40 ± 0.40aAB
200	17.20 ± 1.96cD	56.00 ± 3.85bC	82.80 ± 1.62aB	87.20 ± 2.33aABC	90.80 ± 3.93aAB
250	2.00 ± 0.63dE	23.60 ± 2.79cD	71.20 ± 3.01bC	83.20 ± 3.01aBC	89.20 ± 1.62aBC
300	0.00 ± 0.00dE	10.40 ± 2.99cE	52.80 ± 2.87bD	76.40 ± 1.72aC	83.20 ± 1.85aC
CK	96.40 ± 0.75A	96.40 ± 0.75A	96.40 ± 0.75A	96.40 ± 0.75A	96.40 ± 0.75A

*Note:* Data are presented as mean **±** SE. Means with different letters (lowercase: row; uppercase: column) mean significant difference (*p* < 0.05) according to Tukey's test.

**TABLE 2:** Effects of different age of pupae subjected to X‐ray irradiation on the emergence parameters of *Bactrocera dorsalis* males (one‐way ANOVA).

Emergence rate (%)					
Dose (Gy)	Age (days)				
	5	6	7	8	9
50	79.60 ± 0.98cB	91.60 ± 1.94abAB	96.80 ± 1.36aA	90.00 ± 2.28bAB	95.20 ± 0.49abAB
100	60.80 ± 1.36bC	85.60 ± 2.40aB	90.80 ± 2.42aA	91.60 ± 2.23aAB	92.80 ± 1.20aAB
150	28.80 ± 1.35cD	69.20 ± 3.14bC	91.20 ± 0.80aA	92.40 ± 2.86aAB	93.60 ± 1.17aAB
200	9.60 ± 2.86dE	30.00 ± 2.76cD	77.60 ± 1.94bB	86.40 ± 1.17abABC	90.40 ± 2.99aABC
250	2.80 ± 0.80dF	10.00 ± 1.79cE	52.80 ± 3.38bC	82.40 ± 2.99aBC	88.40 ± 2.14aBC
300	0.00 ± 0.00dG	4.80 ± 0.80cE	30.00 ± 2.76bD	72.00 ± 3.85aC	82.00 ± 2.61aC
CK	95.60 ± 1.17A	95.60 ± 1.17A	95.60 ± 1.17A	95.60 ± 1.17A	95.60 ± 1.17A

*Note:* Data are presented as mean **±** SE. Means with different letters (lowercase: row; uppercase: column) mean significant difference (*p* < 0.05) according to Tukey's test.

### 3.2 Effects of Irradiation on the Flight Capacity of *B. dorsalis*

A two‐way ANOVA showed that both the radiation dose and sex significantly influenced the flight capability of the emerged *B. dorsalis* adults, while their interaction had no notable effect (Table S5). As shown in Figure 1C, exposure of pupae to irradiation doses of 150, 200, 250, and 300 Gy significantly reduced the flight ability of females compared to the control group. For males, a dose‐dependent effect was observed with increasing doses. Compared to control males, significant differences in flight ability were observed in males exposed to X‐ray doses of 200, 250, and 300 Gy during the pupal stage (Figure 1D).

### 3.3 Effects of Irradiation on the Longevity and Fecundity of *B. dorsalis*

According to the two‐way ANOVA, both radiation dose and sex significantly impacted the lifespan of *B. dorsalis* , while their interaction had no significant effect (Table S5). A dose‐dependent effect on longevity was observed in both sexes of *B. dorsalis* when the pupal radiation dose ranged from 50 to 300 Gy (Figure 1E,F). Significant differences in longevity between the control group and treatment groups exposed to 200, 250, and 300 Gy during the pupal stage were observed for both sexes (Figure 1E,F). Regarding survival trends, females exposed to X‐ray doses of 200, 250, and 300 Gy during the pupal stage exhibited a more rapid reduction in survival to 50%, occurring within 27, 19, and 15 days, respectively, compared to 47 days for the control group (Figure 1G). Similarly, males who received pupal radiation doses of 200, 250, and 300 Gy reached 50% survival within 21, 12, and 12 days, respectively, whereas the control group reached 45 days (Figure 1H). Regarding fecundity, pupal radiation posed significantly deteriorated impacts on this index. All radiation treatment resulted in zero eggs laid (Figure 1I).

### 3.4 | Effects of Irradiation on the Sterilization of *B. dorsalis* Males

Two‐way ANOVA results indicate that the radiation dose significantly affects the sterility rate of *B. dorsalis* , while neither male age nor its interaction with the radiation dose showed significant effects (Table S5). As presented in Table 3, complete infertility was recorded in males aged 12, 16, and 20 days when the X‐ray dose reached 70 Gy.

**TABLE 3:** Effects of pupal irradiation on the sterile rate of *Bactrocera dorsalis* males with different ages (one‐way ANOVA).

Sterile rate (%)			
Dose (Gy)	Age (days)		
	12	16	20
50	96.17 ± 1.01b	96.23 ± 0.54b	97.67 ± 0.41b
60	99.40 ± 0.33b	99.17 ± 0.33b	99.60 ± 0.10b
70	100.00 ± 0.00b	100.00 ± 0.00b	100.00 ± 0.00b
80	100.00 ± 0.00b	100.00 ± 0.00b	100.00 ± 0.00b
90	100.00 ± 0.00b	100.00 ± 0.00b	100.00 ± 0.00b
100	100.00 ± 0.00b	100.00 ± 0.00b	100.00 ± 0.00b
CK	16.77 ± 3.31a	16.20 ± 2.86a	18.87 ± 2.58a

*Note:* Data are presented as mean **±** SE. Means with different letters in a column mean significant difference (*p* < 0.05) according to Tukey's test.

### 3.5 Effects of Pupal Irradiation on the Mating Competitiveness of *B. dorsalis* Males

Irradiation doses between 50 and 100 Gy did not notably influence the mating competitiveness of IM (*F*_6, 35_ = 0.571, *p* = 0.751) (Figure 1J).

### 3.6 Alpha and Beta Analysis of Pupal Irradiation on the Gut Bacteria Communities of Emerged *B. dorsalis*

**FIGURE 2:**
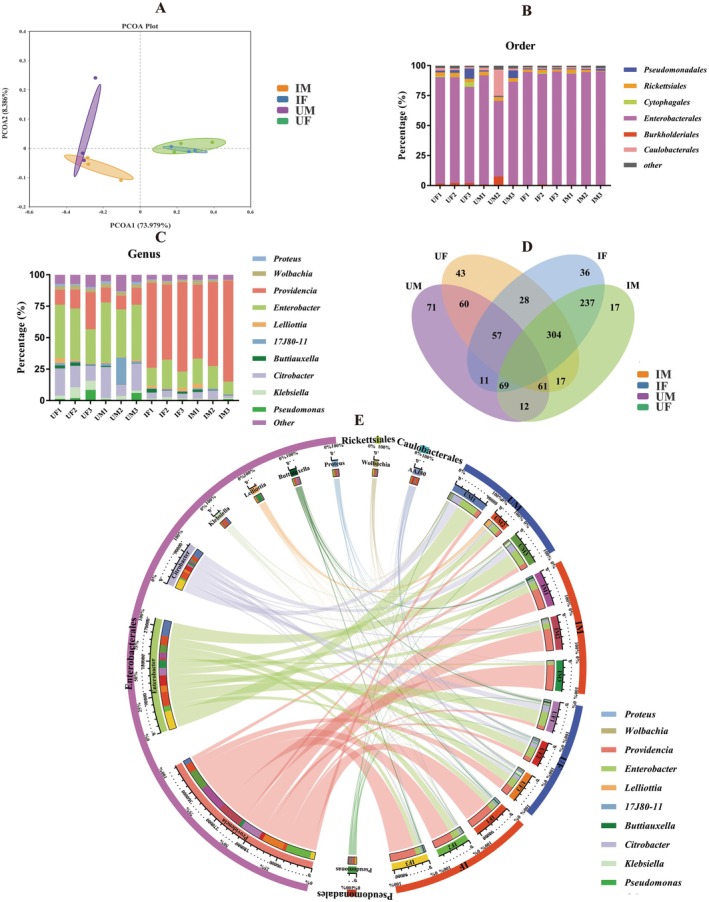
Effect of pupal X‐ray irradiation on the gut bacteria of *Bactrocera dorsalis* . Principal Coordinates Analysis (PCoA) illustrates the clustering patterns and variation among samples (A). Relative abundance of gut bacteria at the order (B) and genus (C) levels. Venn diagrams show the number of differentially abundant bacterial genera between control and treatment groups across sexes (D). A Circos diagram depicting the associations between sample types and the dominant bacterial OTUs (E). The first outer circle represents the sample types and bacterial order names. The second outer circle represents the proportion of each OTU within different samples, as well as the proportion of each sample within the corresponding OTUs. IF, irradiated female; IM, irradiated male; UF, unirradiated female; UM, unirradiated male.

Radiation exposure did not have significant effects on the Shannon and Simpson indices of gut bacterial communities in either sex of *B. dorsalis* . In contrast, the Chao1 and ACE richness indices were significantly affected (Figure S1). The Principal Coordinates Analysis (PCoA) demonstrated a clear separation between the irradiation group and the control group, indicating distinct differences in microbial community composition (PERMANOVA: *F* = 3.8263, *R* ^2^ = 0.5893, *p* < 0.01; PERMDISP: *F* = 0.229, *p* = 0.917) (Figure 2A).

### 3.7 Effects of Pupal Irradiation on the Gut Bacteria Communities

At the order level, the relative abundance of Burkholderiales (*t* = 4.790, df = 4, *p* < 0.01), Enterobacterales (*t* = 3.209, df = 4, *p* < 0.05), and Rickettsiales (*t* = 3.475, df = 4, *p* < 0.05) of females was significantly affected by irradiation. However, no notable difference was observed in the predominant gut bacterial orders between IM and UM (Figure 2B). At the genus level, compared with control females, the relative abundance of *Citrobacter* (*t* = 5.351, df = 4, *p* < 0.01), *Klebsiella* (*t* = 2.925, df = 4, *p* < 0.05), *Wolbachia* (*t* = 3.474, df = 4, *p* < 0.05), *Proteus* (*t* = 5.893, df = 4, *p* < 0.01), and *Enterobacter* (*t* = 3.559, df = 4, *p* < 0.05) in IF was significantly decreased, from 16.97% to 4.57%, 6.15% to 1.36%, 3.12% to 1.78%, 1.41% to 0.43%, and 36.76% to 16.70%, respectively. Conversely, the relative abundance of *Providencia* (*t* = 6.644, df = 4, *p* < 0.01) in IF significantly increased, from 18.75% to 66.00%. In IM, there was a significant decrement in the relative abundance of *Enterobacter* (*t* = 5.957, df = 4, *p* < 0.01) and *Citrobacter* (*t* = 3.029, df = 4, *p* < 0.05), with a reduction from 43.31% to 15.95%, and 18.49% to 4.72%. In contrast, the relative abundance of *Providencia* (*t* = 8.858, df = 4, *p* < 0.01) significantly increased, from 11.92% to 68.53% (Figure 2C). A Venn diagram showed 12 mutual OTUs between UM and IM, while 28 mutual OTUs between UF and IF (Figure 2D). The Circos diagram visually illustrated the differences in dominant genera between irradiated and unirradiated *B. dorsalis* , as well as between females and males, at both the order and genus levels (Figure 2E).

**FIGURE 3:**
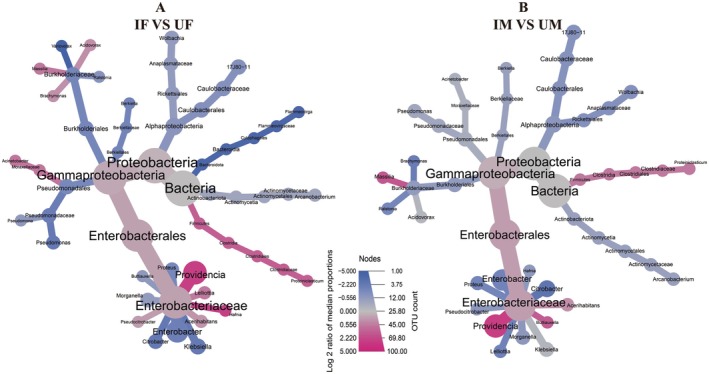
Heat tree diagrams depict differences in bacterial genera between treatment and control groups in *Bactrocera dorsalis* (A: Female; B: Male). Node diameter corresponds to OTU count, with larger nodes indicating higher bacterial abundance and smaller nodes representing lower abundance. Node color represents the median log_2_ ratio of bacterial abundance in irradiated versus control groups, where pink signifies increased abundance (upregulation) and blue indicates decreased abundance (downregulation). IF, irradiated female; IM, irradiated male; UF, unirradiated female; UM, unirradiated male.

The heat tree diagram illustrated the taxonomic differences in bacterial communities of IF versus UF, and IM versus UM. Specifically, it revealed the evolutionary relationships among bacteria and differences in some less prominent bacteria. Obviously, in females, differences were mainly recorded for *Providencia*, *Enterobacter*, *Klebsiella*, *Citrobacter*, *Hafnia*, *Acinetobacter*, *Variovorax*, *Flammeovirga*, and *Pseudomonas* (Figure 3A). In males, the primary differences in bacterial communities were noted for *Enterobacter*, *Citrobacter*, and *Providencia* (Figure 3B).

**FIGURE 4:**
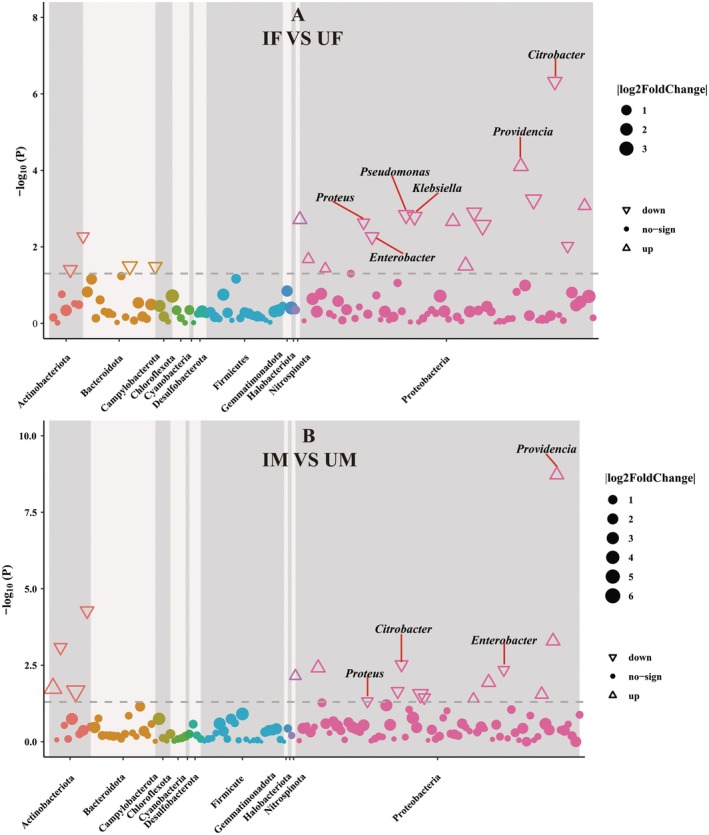
Manhattan plots illustrating the distribution of differentially abundant genera of bacteria (DAGB) between treatment and control groups for female (A) and male (B), respectively. Upward‐pointing triangles represent genera with significantly increased abundance, downward‐pointing triangles indicate significantly decreased abundance, and circles denote genera with no significant difference. The size indicates the number of absolute values of log_2_FoldChange. IF, irradiated female; IM, irradiated male; UF, unirradiated female; UM, unirradiated male.

DESeq2‐based Manhattan plots revealed that irradiation significantly altered gut bacterial composition. In IF versus UF, 20 differentially abundant bacterial genera (DABGs) were identified, including six dominant genera (relative abundance > 1%): *Providencia* (*p* < 0.01), *Pseudomonas* (*p* < 0.01), *Enterobacter* (*p* < 0.01), *Citrobacter* (*p* < 0.01), *Proteus* (*p* < 0.01), and *Klebsiella* (*p* < 0.01) (Figure 4A). In IM versus UM, 17 DABGs were detected, with four dominant genera: *Providencia* (*p* < 0.01), *Enterobacter* (*p* < 0.01), *Citrobacter* (*p* < 0.01), and *Proteus* (*p* < 0.05) (Figure 4B).

### 3.8 Correlation Between Dominant Bacterial Genera

**FIGURE 5:**
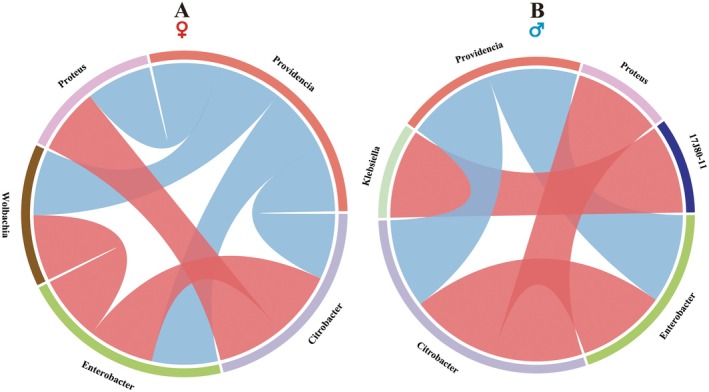
Effect of pupal X‐ray irradiation on the correlation relationship between dominant gut bacterial genera (relative abundance > 1%) in *Bactrocera dorsalis* (A: Female; B: Male) based on Pearson's correlation coefficient. Red and blue indicate significant positive and negative correlations, respectively (*p* < 0.05).

As shown in Figure 5A, among the dominant bacterial genera, significant inverse correlations were recorded in the gut community of females between *Citrobacter* and *Providencia* (*r* = −0.992, *p* < 0.01), *Enterobacter* and *Providencia* (*r* = −0.972, *p* < 0.01), *Wolbachia* and *Providencia* (*r* = −0.939, *p* < 0.01), and between *Proteus* and *Providencia* (*r* = −0.919, *p* < 0.01). Meanwhile, significant positive correlations were recorded between *Enterobacter* and *Citrobacter* (*r* = 0.964, *p* < 0.01), *Enterobacter* and *Wolbachia* (*r* = 0.963, *p* < 0.01), and between *Proteus* and *Citrobacter* (*r* = 0.930, *p* < 0.01). For males, significant negative correlations were observed between *Enterobacter* and *Providencia* (*r* = −0.979, *p* < 0.01), and between *Citrobacter* and *Providencia* (*r* = −0.846, *p* < 0.05). Except for these, all other correlations in the figure were significantly positive (Figure 5B).

### 3.9 Effects of Irradiation on the Transcriptome of *B. dorsalis*

**FIGURE 6:**
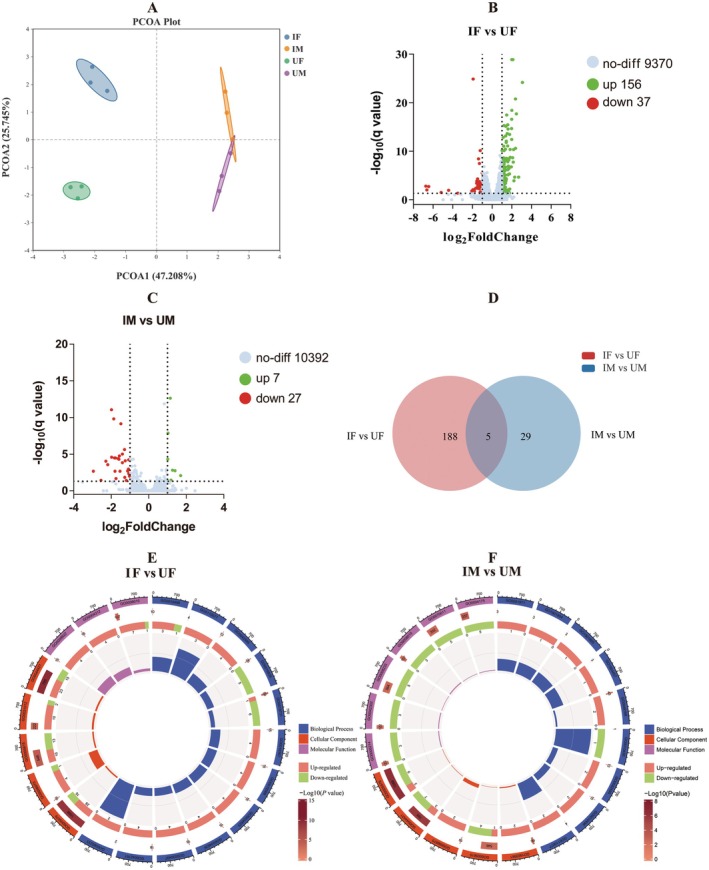
Effect of pupal X‐ray irradiation on the transcriptome of *Bactrocera dorsalis*. Principal Coordinates Analysis (PCoA) illustrates sample clustering and variation (A). Venn diagram depicts the number of differentially expressed genes (DEGs) in the two comparisons (B). Volcano plots of DEGs between treatment and control groups for female (C) and male (D), respectively, with red and blue points representing upregulated and downregulated DEGs. Gene Ontology (GO) enrichment analysis of DEGs between treatment and control groups for female (E) and male (F). The first outer circle represents the top 20 enriched GO terms, with different colors indicating distinct ontologies. The second outer circle displays the total number of associated genes and the *p* value for each GO term. The third outer circle, with red and green segments, represents the number of upregulated and downregulated DEGs, respectively. The fourth outer circle illustrates the enrichment factor for each GO term. IF, irradiated female; IM, irradiated male; UF, unirradiated female; UM, unirradiated male.

PCoA analysis showed distinct separation among the four experimental groups (IF, UF, IM, UM), reflecting both sex‐ and irradiation‐induced transcriptomic divergence. The overall group differences were statistically significant (PERMANOVA: *F* = 5.0863, *R* ^2^ = 0.6560, *p* < 0.001), and not attributable to within‐group variance (PERMDISP: *F* = 0.2541, *p* = 0.842) (Figure 6A). The DEGs were visualized using a volcano plot. A total of 193 DEGs were identified in IF versus UF, including 156 up‐regulated genes and 37 down‐regulated genes (Figure 6B). For males, a total of 34 DEGs were identified in IM versus UM, including 7 up‐regulated genes and 27 down‐regulated genes (Figure 6C). Five DEGs were selected for RT‐qPCR validation in both males and females. The RT‐qPCR analysis confirmed that the expression profiles of these genes were highly consistent with those identified through RNA‐seq, reinforcing the trustworthiness and precision of the transcriptomic findings (Figure S2). In addition, five common DEGs were identified between the IF versus UF and IM versus UM, namely, *LOC105230251* (*Pla1a*), *LOC109579239* (*OB56A*), *LOC115066471* (*Arc1*), *LOC125776279* (lysosomal acid glucosylceramidase‐like), and *LOC105230524* (activity‐regulated cytoskeleton associated protein 2‐like) (Figure 6D).

Gene Ontology (GO) enrichment analysis revealed that the top 20 enriched GO terms in IF versus UF were distributed as follows: 12 terms for biological process, 5 terms for cellular component, and 3 terms for molecular function (Figure 6E). For IM versus UM, the distribution was 9 terms for biological process, 5 terms for cellular component, and 6 terms for molecular function (Figure 6F). Overall, 6 mutual terms were identified in both the comparisons of IF versus UF and IM versus UM. These terms included GO:0044421 (extracellular region part), GO:0098975 (postsynapse of neuromuscular junction), GO:0005615 (extracellular space), GO:0004252 (serine‐type endopeptidase activity), GO:0010496 (intercellular transport), and GO:0042595 (behavioral response to starvation) (Figure 6E,F).

### 3.10 Correlation Between DABGs and DEGs

**FIGURE 7:**
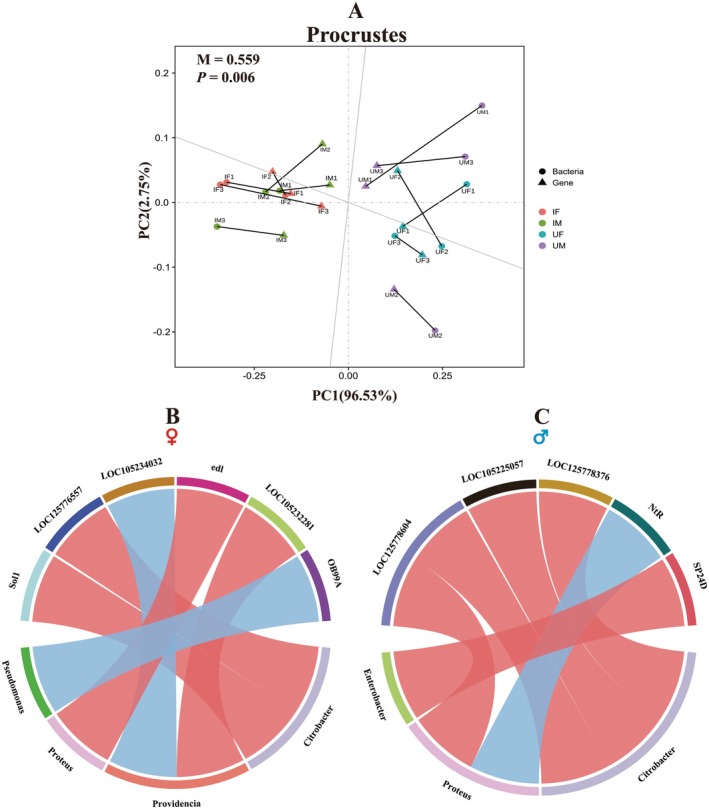
Integrated analysis of differently abundant bacterial genera and differently expressed genes. Procrustes analysis depicts the similarity between bacterial samples and transcriptome samples (A). Correlation relationship between differently changed bacterial genera and differently expressed genes according to Pearson's correlation coefficient (B: Female; C: Male). Red and blue indicate significant positive and negative correlations, respectively, with significance thresholds of *p* = 0.01 for females and *p* = 0.05 for males. IF, irradiated female; IM, irradiated male; UF, unirradiated female; UM, unirradiated male.

Procrustes analysis revealed significant similarities between bacterial and transcriptome samples from the same treatment, with irradiated samples showing notable divergence from unirradiated ones (Figure 7A). In females, significant correlations (*p* < 0.01) were identified between *Proteus* and *LOC105230647* (*edl*) (*r* = 0.999), *Pseudomonas* and *LOC105231039* (*OB99A*) (*r* = 0.999), *Providencia* and both *LOC105234032* (kunitz‐type U15‐theraphotoxin‐Hs1g) and *LOC105232281* (mantle protein) (*r* = 0.999), as well as *Citrobacter* and both *LOC105225826* (*Sol1*) and *LOC125776557* (*r* = 0.999) (Figure 7B). In males, *Citrobacter* showed significant positive correlations with *LOC125778604* (*r* = 0.999, *p* < 0.01), *LOC105225057* (lysosomal aspartic protease) (*r* = 0.999, *p* < 0.05), and *LOC125778376* (jerky protein homolog‐like) (*r* = 0.998, *p* < 0.05). *Proteus* was positively correlated with *LOC125778604* (*r* = 0.998, *p* < 0.05), but negatively correlated with *LOC105230182* (*NtR*) (*r* = −0.998, *p* < 0.05). Additionally, *Enterobacter* exhibited a significant positive correlation with *LOC105227477* (*SP24D*) (*r* = 0.998, *p* < 0.05) (Figure 7C).

## 4 Discussion

Our results indicated that X‐ray dose, the age of pupae at irradiation, and their interaction negatively affected the eclosion parameters of *B. dorsalis* GSS. Irradiation of 8‐day‐old pupae notably suppressed the flight ability, lifespan, and fecundity in emerging adults. Furthermore, when the irradiation dose exceeded 70 Gy, the male sterility rate reached 100%, although their mating competitiveness remained unimpaired. X‐ray irradiation induced notable shifts in the gut microbiota composition of *B. dorsalis* , marked by a reduction in the abundance of *Enterobacter*, *Citrobacter*, and *Proteus*, accompanied by an enrichment of *Providencia*. Additionally, broad correlations among dominant bacterial genera were observed. Transcriptomic analysis indicated that irradiation exerted remarkable impacts on gene expression in both male and female adults. Specifically, 100 and 34 differentially expressed genes (DEGs) were detected in females and males, respectively. Gene Ontology (GO) enrichment analysis showed that 6 GO terms were enriched in both sexes. Moreover, broad correlations between specific differentially abundant bacterial genera (DAGBs) and DEGs were observed, further supporting the interaction between intestinal microbiome and host gene expression.

The constraints of gamma rays have hindered the further development and adoption of SIT in agricultural pest control (Mastrangelo et al. 2010). To overcome the limitations of traditional gamma radiation, more recent studies have explored the utilization of X‐ray as an alternative radiation source for inducing sterility in target insects and evaluating its practical feasibility (Huang et al. 2024; Yun et al. 2016; Zhao et al. 2022). This study found that X‐ray significantly affected the emergence parameters of *B. dorsalis* pupae at various ages, showing a clear age‐dependent effect when irradiation dose ranged from 100 to 300 Gy. This result aligns with a prior study, which indicated the emergence of *B. dorsalis* subjected to 100 Gy X‐ray radiation increased as the pupal ages (Fezza et al. 2021). Studies on *Zeugodacus tau* (Diptera: Tephritidae) and *Z. cucurbitae* also showed a positive correlation between radiation resistance and the developmental stage of pupae (Wadud et al. 2005; Du et al. 2016). Given the differences in irradiation sensitivity between male and female pupae at various ages, as well as the stability of emergence rhythms in irradiated pupae during transportation, this study, consistent with previous research (Yusof et al. 2019; Fezza et al. 2021), selected 8‐day‐old pupae as the optimal age for irradiation, and conducted subsequent experiments.

Further evaluation of the quality of irradiated adults revealed significant effects on flight ability, fecundity, and longevity. These findings are consistent with those of Yusof et al. (2019) who studied the effects of Cs^137^ irradiation on *B. dorsalis* , showing a remarkable decrement in lifespan as radiation doses increased, along with a marked suppression of oviposition and reproductive organ development in females at higher doses. In male insects, the extent of germ cell damage induced by irradiation directly influences the sterility of eggs laid by females after mating (Vreysen and Robinson 2011). Additionally, a key factor affecting the effectiveness of SIT in the field is maintaining a balance between male mating competitiveness and egg sterility induction (Barry et al. 2004; Toledo et al. 2004). Our results indicated that at an irradiation dose of 70 Gy, males achieved complete sterility without a significant reduction in mating competitiveness. It is worth noting that this dose is slightly higher than the Co^60^ radiation (60 Gy) reported in a previous study (Zahan et al. 2016), which may be due to the strategy of periodically introducing wild‐type genes in this study, or the sensitivity of *B. dorsalis* to different radiation sources.

The intestinal microbiome is indispensable for the adaptive response to external environmental stresses (low temperatures, pathogens, pesticides, and radiation) of tephritid species (Cheng et al. 2017; Hamden et al. 2020; Raza, Wang, et al. 2020; Raza, Yao, et al. 2020; Haider et al. 2025). Consequently, evaluating the impact of irradiation on gut communities is essential for understanding the functional role of intestinal microbiota in the host's radioprotective mechanisms. X‐ray irradiation elicited pronounced alterations in the gut microbiota composition of *B. dorsalis* , characterized by significant reductions in the abundance of *Enterobacter*, *Citrobacter*, and *Proteus*, alongside a marked enrichment of *Providencia*. Previous research indicated that a 50 Gy Co^60^ irradiation of *B. dorsalis* pupae increased gut microbiota diversity, accompanied by a decrease in *Orbus* and *Lactococcus*, and an increase in *Lactobacillus* and *Morganella* (Stathopoulou et al. 2019). Additionally, Cai et al. (2018a, 2018b) and Cai et al. (2018) reported that a 100 Gy Co^60^ irradiation of *B. dorsalis* pupae remarkably affected the diversity of gut microbiota, with a notable decrease in the relative abundance of *Citrobacter koseri* , *Klebsiella michiganensis*, and *Enterobacter soli* . These discrepancies are primarily attributed to differences in the intestinal microbiota composition of *B. dorsalis* across various regions and strains, as well as the varying sensitivity of gut microbiota to different radiation sources. It is well established that most members of *Enterobacter* and *Citrobacter* maintain symbiotic relationships with tephritid species, directly or indirectly influencing various life processes, including development, nutrient uptake, immune defense, and male competitiveness (Raza, Wang, et al. 2020; Raza, Yao, et al. 2020; Hafsi and Delatte 2023). Conversely, certain members of *Providencia* have been identified as opportunistic pathogens, accelerating mortality in hosts, including *B. dorsalis* , *Anastrepha ludens* (Diptera: Tephritidae), and *Ceratitis capitata* (Diptera: Tephritidae) (Guerfali et al. 2018; Gu et al. 2023; Salas et al. 2023). Therefore, we hypothesize that the dynamic changes in the gut microbiota abundance of *B. dorsalis* may be partly due to irradiation‐induced disruption of the host's immune system and beneficial microbiota, which subsequently triggers the rapid proliferation of opportunistic pathogens.

Our findings demonstrated that X‐ray significantly influenced the transcriptomes of both male and female adults. Enrichment analysis of the DEGs revealed several shared GO terms between the sexes, including GO:0042595 (behavioral response to starvation), GO:0044421 (extracellular region part), GO:0005615 (extracellular space), and GO:0010496 (intercellular transport). High‐dose irradiation is well known for not only causing chromosome damage but also accelerating apoptosis and necrosis (Li et al. 2019). Furthermore, when insects encounter adverse environmental stressors, they typically consume large amounts of nutrients and strategically increase their metabolic activity to maintain physiological balance (Colinet and Boivin 2011; Lin et al. 2024). Therefore, we hypothesize that the activation of starvation response, along with extracellular and intercellular pathways, may be a strategy employed by *B. dorsalis* pupae to mitigate radiation‐induced cellular damage by consuming large amounts of nutrients to maintain cellular activities, particularly repair processes, thereby reducing cell death. Certainly, the activation of starvation pathways may also be attributed to the dramatic proliferation of the opportunistic pathogen *Providencia*, which competes intensively with the host for available nutrients. Furthermore, irradiation led to the activation of pathways related to serine (GO:0004252). Serine‐related proteins play crucial roles in biological processes such as cellular responses, apoptosis, cellular proliferation, and survival (Cross et al. 2000). Moreover, we observed a positive enrichment for GO:0098975 (postsynapse of neuromuscular junction), which we speculate may be associated with adaptive changes at the neuromuscular junction in response to irradiation stress.

Overall, our findings suggest that X‐ray irradiation adversely affected the emergence parameters, flight ability, lifespan, and fecundity of *B. dorsalis* . However, at an irradiation dose of 100 Gy, the quality of sterile male flies was preserved. Additionally, X‐ray irradiation altered the intestinal bacterial composition and the transcriptomes of adults. Future studies should investigate whether the interaction between the gut microbiome and host gene expression contributes to the response of *B. dorsalis* to radiation stress. Our research provides important theoretical support for the improvement of SIT of *B. dorsalis*.

## Conflicts of Interest

The authors declare no conflicts of interest.

## Supporting information


**Table S1:** Primer list of this experiment.
**Table S2:** Effects of pupal age, sex, irradiation dose, and their interaction on the emergence parameters of *Bactrocera dorsalis* adults (three‐way ANOVA).
**Table S3:** Effects of different age of pupae subjected to X‐ray irradiation on the partial adult rate of *Bactrocera dorsalis* males (one‐way ANOVA).
**Table S4:** Effects of different age of pupae subjected to X‐ray irradiation on the deformed adult rate of *Bactrocera dorsalis* males (one‐way ANOVA).
**Table S5:** Effects of pupal age, sex, irradiation dose, and their interaction on the emergence parameters of *Bactrocera dorsalis* adults (Three‐way ANOVA).
**Figure S1:** Effects of X‐ray irradiation on the richness index (A) and diversity index (B) of the gut bacteria of *Bactrocera dorsalis* .
**Figure S2:** RT‐qPCR validation of expression levels of genes in the RNA‐seq data.

## Data Availability

Both the 16S rRNA sequencing data and the transcriptomic datasets have been submitted to the NCBI Sequence Read Archive (SRA) and are available under BioProject accession numbers PRJNA1290855 and PRJNA1291910. Data will be made available on request.
