# Supplementary material for: Effects of X‐Ray Irradiation on the Biological Parameters, Gut Microbiota, and Gene Expression of Bactrocera dorsalis: Implications for the Sterile Insect Technique
Source: Evol Appl. 2025 Oct 2;18(10):e70158. doi: 10.1111/eva.70158 (PMC12489746; doi:10.1111/eva.70158)
Supplement: Supplementary file 1 — Table S1: Primer list of this experiment. Table S2: Effects of pupal age, sex, irradiation dose, and their interaction on the emergence parameters of Bactrocera dorsalis adults (three‐way ANOVA). Table S3: Effects of different age of pupae subjected to X‐ray irradiation on the partial adult rate of Bactrocera dorsalis males (one‐way ANOVA). Table S4: Effects of different age of pupae subjected to X‐ray irradiation on the deformed adult rate of Bactrocera dorsalis males (one‐way ANOVA). Table S5: Effects of pupal age, sex, irradiation dose, and their interaction on the emergence parameters of Bactrocera dorsalis adults (Three‐way ANOVA). Figure S1: Effects of X‐ray irradiation on the richness index (A) and diversity index (B) of the gut bacteria of Bactrocera dorsalis . Figure S2: RT‐qPCR validation of expression levels of genes in the RNA‐seq data. [file EVA-18-e70158-s001.doc]

**Table S1** Primer list of this experiment

| Gene ID | Primer sequence ( 5 ' -3 ' ) |
| --- | --- |
| LOC105222655 | F: CGCAACACGCAGCTATCAAA |
|  | R: AGAAGTTACTGCGGGTGGTG |
| *LOC105231049* | F: TTCATGTCGGCCCATTTCCA |
|  | R: CTTCCAGACGGGTCCTTCAC |
| *LOC125776557* | F: GCACGCGGTTAATCCCAATC |
|  | R: AATCATTTGTTGCCCGCTCG |
| *LOC105230524* | F: TTCGCGATCACTTCTCACCC |
|  | R: CGCACGCTTCTGACAAACAA |
| *LOC115066471* | F: TTCGCGATCACTTCTCACCC |
|  | R: CGCACGCTTCTGACAAACAA |
| *LOC105226014* | F: TTGGTACGCGGTCAGTTTGT |
|  | R: ATTAGAGCCGCCAATGCTGT |
| *LOC105227473* | F: GTGGTTGAAGCCACGCAAAA |
|  | R: TAGACACAATGTGCTGCGGT |
| *LOC105223567* | F: ACGCACCTTCGGTTTCTTCT |
|  | R: CGATTGAGTATGCGACCCGA |
| *α-Tubin* | F: CGCATTCATGGTTGATAACG |
|  | R: GGGCACCAAGTTAGTCTGGA |

**Table S2** Effects of pupal age, sex, irradiation dose, and their interaction on the emergence parameters of *B. dorsalis* adults (Three-way ANOVA)

| Emergence parameters | Factors | *df* | *F* | *P* |
| --- | --- | --- | --- | --- |
| Emergence rate | A | 4, 300 | 882.958 | <0.01 |
|  | B | 1, 300 | 49.125 | <0.01 |
|  | C | 5, 300 | 577.205 | <0.01 |
|  | A × B | 4, 300 | 6.969 | <0.01 |
|  | A × C | 20, 300 | 97.393 | <0.01 |
|  | B × C | 5, 300 | 1.017 | 0.408 |
|  | A × B × C | 20, 300 | 3.265 | <0.01 |
| Partial emergence rate | A | 4, 300 | 114.591 | <0.01 |
|  | B | 1, 300 | 0.005 | 0.943 |
|  | C | 5, 300 | 68.553 | <0.01 |
|  | A × B | 4, 300 | 1.184 | <0.01 |
|  | A × C | 20, 300 | 8.104 | <0.01 |
|  | B × C | 5, 300 | 4.047 | <0.01 |
|  | A × B × C | 20, 300 | 3.085 | <0.01 |
| Deformed adult rate | A | 4, 300 | 18.099 | <0.01 |
|  | B | 1, 300 | 1.594 | 0.208 |
|  | C | 5, 300 | 9.378 | <0.01 |
|  | A × B | 4, 300 | 0.254 | 0.907 |
|  | A × C | 20, 300 | 6.234 | <0.01 |
|  | B × C | 5, 300 | 1.304 | 0.263 |
|  | A × B × C | 20, 300 | 1.716 | <0.05 |

A: Pupal age; B: Sex; C: Irradiation dose

**Table S3** Effects of different age of pupae subjected to X-ray irradiation on the partial adult rate of *Bactrocera dorsalis* males (one-way ANOVA)

| **Partial Emergence Rate (%)** | | | | | | | | | | |
| --- | --- | --- | --- | --- | --- | --- | --- | --- | --- | --- |
| Sex | Dose (Gy) | Day before emergence | | | | | | | | |
|  |  | 5 | 6 | | | 7 | | 8 | 9 | |
| Male | 50 | 4.80±1.62aAB | 1.60±0.75abA | | | 0.40±0.40bA | | 3.60±0.75AaB | 0.40±0.40bA | |
|  | 100 | 20.00±1.10aC | 3.60±1.17bA | | | 3.20±1.20bB | | 2.00±0.63bAB | 2.00±0.63bA | |
|  | 150 | 40.80±1.85aD | 16.40±1.32bB | | | 2.80±0.49cB | | 2.80±1.02cAB | 2.00±0.63cA | |
|  | 200 | 54.00±1.79aE | 35.20±3.01bC | | | 9.20±0.80cC | | 3.20±1.02dAB | 2.80±1.02dA | |
|  | 250 | 27.60±2.64bCD | 59.60±3.60aD | | | 23.60±1.17bD | | 4.80±1.36cAB | 2.80±0.80cA | |
|  | 300 | 8.00±1.90cB | 51.60±2.86aD | | | 37.60±2.40bE | | 8.80±1.85cB | 1.60±0.40dA | |
| CK |  | 2.00±0.89A | | 2.00±0.89A | 2.00±0.89AB | | 2.00±0.89A | | | 2.00±0.89A |
| Female | 50 | 3.20±0.80aAB | | 3.20±1.36aA | 3.20±0.49aAB | | 1.20±0.49abA | | | 0.00±0.00bA |
|  | 100 | 6.80±1.76aB | | 4.80±1.02aA | 2.80±0.80aA | | 2.00±0.63aA | | | 2.80±1.02aAB |
|  | 150 | 28.80±2.33aC | | 4.80±1.02bA | 3.60±1.94bcAB | | 4.00±1.94bAB | | | 0.40±0.40cA |
|  | 200 | 62.00±2.53aD | | 24.40±3.43bB | 8.80±1.50cBC | | 2.40±0.75dAB | | | 2.40±1.47dAB |
|  | 250 | 63.20±1.85aD | | 56.00±1.79aC | 9.20±0.80bC | | 7.20±1.02bBC | | | 1.20±0.49cAB |
|  | 300 | 36.40±1.17bC | | 75.60±4.45aD | 25.60±1.47bD | | 13.60±1.47cC | | | 6.80±2.24cB |
|  |  | 1.20±0.49A | | 1.20±0.49A | 1.20±0.49A | | 1.20±0.49A | | | 1.20±0.49AB |

Data are presented as mean **±** SE. Means with different letters (lowercase: row; uppercase: column) mean significant difference (*P* < 0.05) according to Tukey's test

For the partial emergence rate, the only factor that did not show a significant effect was sex (Table S1). Five-day-old female (200 Gy: *P* < 0.01; 250 Gy: *P* < 0.01; 300 Gy: *P* < 0.01) and male pupae (200 Gy: *P* < 0.01; 250 Gy: *P* < 0.01; 300 Gy: *P* < 0.01) exposed to irradiation doses greater than 200 Gy exhibited significantly higher values compared to the control group. A similar trend was observed in 6-day-old female (200 Gy: *P* < 0.01; 250 Gy: *P* < 0.01; 300 Gy: *P* < 0.01) and male pupae (200 Gy: *P* < 0.01; 250 Gy: *P* < 0.01; 300 Gy: *P* < 0.01), as well as in 7-day-old female (200 Gy: *P* < 0.01; 250 Gy: *P* < 0.01; 300 Gy: *P* < 0.01) and male pupae (200 Gy: *P* < 0.01; 250 Gy: *P* < 0.01; 300 Gy: *P* < 0.01). 8-day-old pupae exposed to a dose of 300 Gy exhibited a significantly higher partial emergence rate than the control group (female: *P* < 0.01; male: *P* < 0.05). For 9-day-old pupae, no significant difference was observed when compared to the control. Moreover, at the same radiation dose, 9-day-old pupae showed the lowest partial emergence rate compared to other pupal ages, except for those treated with 100 Gy. In contrast, 5-day-old male pupae exhibited the highest partial emergence rate with radiation doses ranging from 50 to 200 Gy, while female pupae showed the highest rate within the 50 to 250 Gy range. At a radiation dose of 300 Gy, 6-day-old pupae displayed the highest partial emergence rate (Table S2).

**Table S4** Effects of different age of pupae subjected to X-ray irradiation on the deformed adult rate of *Bactrocera dorsalis* males (one-way ANOVA)

| **Deformed Adult Rate (%)** | | | | | | | | | | |
| --- | --- | --- | --- | --- | --- | --- | --- | --- | --- | --- |
| Sex | Dose (Gy) | Day before emergence | | | | | | | | |
|  |  | 5 | 6 | | | 7 | | 8 | 9 | |
| Male | 50 | 2.00±0.89aAB | 1.60±0.40aA | | | 0.40±0.40aA | | 0.80±0.49aA | 0.00±0.00aA | |
|  | 100 | 4.80±0.80aBC | 1.60±0.40abA | | | 0.80±0.49bA | | 2.00±0.00abAB | 1.20±0.49bA | |
|  | 150 | 9.60±2.56aC | 4.80±1.37abAB | | | 1.20±0.49bA | | 1.20±0.49bA | 1.20±0.49bA | |
|  | 200 | 1.60±0.75bAB | 10.00±1.55aB | | | 8.00±1.41aB | | 0.40±0.40bA | 0.00±0.00bA | |
|  | 250 | 1.20±0.49bAB | 2.80±1.02bA | | | 10.00±1.67aB | | 2.00±0.63bAB | 0.80±0.49bA | |
|  | 300 | 0.80±0.49bAB | 3.20±1.62bA | | | 12.80±2.58aB | | 5.60±1.60abB | 0.40±0.40bA | |
| CK |  | 0.40±0.40A | | 0.40±0.40A | 0.40±0.40A | | 0.40±0.40A | | | 0.40±0.40A |
| Female | 50 | 2.00±0.89aAB | | 0.80±0.49aA | 1.20±0.80aA | | 0.80±0.49aA | | | 0.40±0.40aA |
|  | 100 | 3.20±1.02aBC | | 1.20±0.49aA | 1.20±0.49aA | | 1.60±0.75aA | | | 1.20±0.49aA |
|  | 150 | 7.20±1.02aB | | 4.80±0.49abB | 2.40±0.75bcA | | 2.00±0.63bcA | | | 0.80±0.49cA |
|  | 200 | 4.00±0.63bBC | | 12.00±2.00aC | 2.80±0.80bcA | | 3.20±0.80bcA | | | 0.80±0.49cA |
|  | 250 | 1.60±0.75bcAB | | 7.20±1.02aBC | 9.20±0.80aB | | 4.40±0.98abA | | | 0.40±0.40cA |
|  | 300 | 0.00±0.00cA | | 4.80±1.02bB | 15.20±1.50aB | | 2.00±1.10bcA | | | 2.80±0.80bA |
|  |  | 0.80±0.49AB | | 0.80±0.49A | 0.80±0.49A | | 0.80±0.49A | | | 0.80±0.49A |

Data are presented as mean **±** SE. Means with different letters (lowercase: row; uppercase: column) mean significant difference (*P* < 0.05) according to Tukey's test

Sex, along with its interactions with pupal age and irradiation dose, had a notable influence on the deformed adult rate (Table S1). Five-day-old pupae irradiated with a dose of 150 Gy (female: *P* < 0.01; male: *P* < 0.01), 6-day-old pupae irradiated with 200 Gy (female: *P* < 0.01; male: *P* < 0.01), and 7-day-old pupae irradiated with 250 Gy (female: *P* < 0.01; male: *P* < 0.01) and 300 Gy (female: *P* < 0.01; male: *P* < 0.01) exhibited significantly higher rates compared to the control group. Radiation did not significantly affect the deformed adult rate in 9-day-old pupae. Additionally, the highest deformed adult rate was observed in 7-day-old pupae irradiated with 300 Gy (Table S3).

**Table S5** Effects of pupal age, sex, irradiation dose, and their interaction on the emergence parameters of *B. dorsalis* adults (Three-way ANOVA)

| Emergence parameters | Factors | *df* | *F* | *P* |
| --- | --- | --- | --- | --- |
| Flight ability | Dose | 6, 84 | 49.435 | <0.01 |
|  | Sex | 1, 84 | 7.300 | <0.01 |
|  | Dose ×Sex | 6, 84 | 1.453 | 0.207 |
| Longevity | Dose | 6, 280 | 114.591 | <0.01 |
|  | Sex | 1, 280 | 0.005 | <0.01 |
|  | Dose ×Sex | 6, 280 | 68.553 | 0.997 |
| Sterile rate | Dose | 6, 126 | 2206.590 | <0.01 |
|  | Age | 2, 126 | 0.613 | 0.613 |
|  | Dose × Age | 12, 126 | 0.247 | 0.247 |





**Figure S1** Effects of X-ray irradiation on the richness index (A) and diversity index (B) of the gut bacteria of *B. dorsalis*. *, and n.s. denote statistical significance levels according to independent samples t-test, where * represents *P* < 0.05, and n.s. indicates *P* > 0.05.


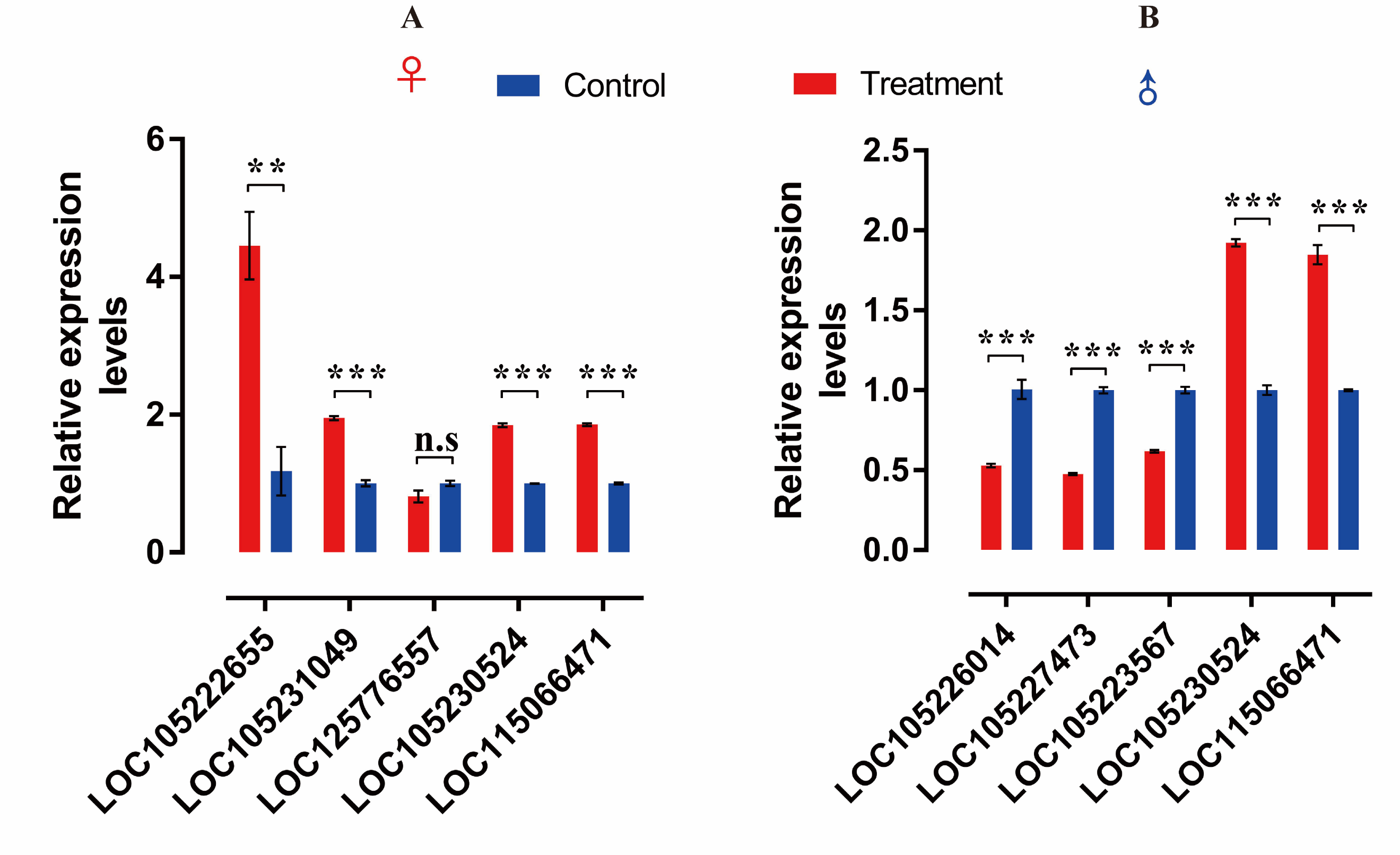


**Figure S2** RT-qPCR validation of expression levels of genes in the RNA-seq data. Data are presented as mean±SE. **, ***, and n.s. denote statistical significance levels according to Tukey’s test, where ** represents *P* < 0.01, *** represents *P* < 0.001, and n.s. indicates *P* > 0.05.
